# Early gross motor skills predict the subsequent development of language in children with autism spectrum disorder

**DOI:** 10.1002/aur.1587

**Published:** 2015-12-22

**Authors:** Rachael Bedford, Andrew Pickles, Catherine Lord

**Affiliations:** ^1^ Biostatistics DepartmentInstitute of Psychiatry, King's College London; ^2^ Department of PsychiatryCenter for Autism and the Developing Brain, Weill Cornell Medical CollegeNew York

**Keywords:** walking, language development, autism spectrum disorder

## Abstract

**Background**: Motor milestones such as the onset of walking are important developmental markers, not only for later motor skills but also for more widespread social‐cognitive development. The aim of the current study was to test whether gross motor abilities, specifically the onset of walking, predicted the subsequent rate of language development in a large cohort of children with autism spectrum disorder (ASD). **Methods**: We ran growth curve models for expressive and receptive language measured at 2, 3, 5 and 9 years in 209 autistic children. Measures of gross motor, visual reception and autism symptoms were collected at the 2 year visit. In Model 1, walking onset was included as a predictor of the slope of language development. Model 2 included a measure of non‐verbal IQ and autism symptom severity as covariates. The final model, Model 3, additionally covaried for gross motor ability. **Results**: In the first model, parent‐reported age of walking onset significantly predicted the subsequent rate of language development although the relationship became non‐significant when gross motor skill, non‐verbal ability and autism severity scores were included (Models 2 & 3). Gross motor score, however, did remain a significant predictor of both expressive and receptive language development. **Conclusions**: Taken together, the model results provide some evidence that early motor abilities in young children with ASD can have longitudinal cross‐domain influences, potentially contributing, in part, to the linguistic difficulties that characterise ASD. ***Autism Res*** *2016, 9: 993–1001*. © 2015 The Authors Autism Research published by Wiley Periodicals, Inc. on behalf of International Society for Autism Research

## Introduction

Early in development, gross motor skills such as rolling over, crawling and walking, play an important role in enabling infants to interact with the world. While these “motor milestones” are widely recognised as important developmental markers, more recently research has begun to examine the links between motor skills and more general social‐cognitive development [see Leonard & Hill, 2015 for a review]. Given the emerging findings of early motor impairments in young children with an autism spectrum disorder (ASD) as well as in infants at familial high‐risk [see Bhat, Landa, & Galloway, 2011; Leonard, Elsabbagh, & Hill, 2014; Rogers, 2009], understanding the relationship between onset of gross motor (GM) milestones and later social communication abilities in these children is of particular interest.

### Motor Skills and Language in Typical Development

Using questionnaires and standardised measures of language and motor skills in typically developing 21 months old, Alcock and Krawczyk [2010] found concurrent links between parent‐reported gross and fine motor skills and language development, although they found no relationship between these abilities on the Bayley Scales of Intellectual Development [Bayley, 1993]. Wang, Lekhal, Aarø and Schjolberg [2014] analysed data from a longitudinal cohort study and found that both parent‐reported motor and communication skills at 18 months were significant predictors of subsequent 3‐year communication ability. However, using only parent‐report questionnaires may be problematic as there is shared variance arising from the fact that the same person reports both motor and language ability. This could explain why Alcock and Krawczyk [2010] did not replicate their findings when using a standardised developmental assessment.

Evidence that specific motor skills precede and predict the later development of social communication has also been provided by several studies using direct observational methods. In typically developing infants, increased rhythmical arm movements have been observed prior to the onset of canonical babbling [e.g. Ejiri, 1998; Iverson, Hall, Nickel, & Wozniak, 2007]. Canonical babbling involves the rhythmical repetition of consonant‐vowel syllables, e.g. ba‐ba‐ba, and Iverson [2010] suggests that the preceding arm movements may play a causal role in the development of this form of babbling through multisensory feedback.

### Walking and Language in Typical Development

Learning to walk marks another important developmental milestone, which is typically achieved around 12 months of age [Adolph & Robinson, 2013; Onis, 2006]. The ability to move independently while having the hands free to gesture and to carry objects represents a key change from earlier sitting and crawling milestones. Indeed, several studies have shown that as well as being a motor milestone, walking also reflects a shift in socio‐cognitive development. In comparison to age‐matched crawling infants, independent walkers show increased vocalisations, directed gestures and social interaction bids [Clearfield, 2011; Clearfield, Osborne, & Mullen, 2008], although of course such relationships are likely to be bidirectional [Karasik, Tamis‐LeMonda, & Adolph, 2011]. The onset of walking has also been linked to emotional changes, with walking infants showing increased elation together with greater levels of wilfulness [Biringen, Emde, Campos, & Appelbaum, 1995; Mahler et al., 1975]. Recently, the shift from crawling to walking has been shown to predict both receptive and expressive vocabulary in typically developing infants [Walle & Campos, 2014]. This relationship could be explained, in part, by a change in the nature of infants' communication bids following walking onset, which in turn alters the verbal responses from mothers [Karasik, Tamis‐LeMonda, & Adolph, 2014].

While the upright posture increases the infant's visual field, and frees the child's hands, it may be change in learning opportunities and associated brain development that results from walking onset, rather than the postural position *per se* which underlies the observed shift in social interaction behaviors. Clearfield [2011] found that, for prewalking infants, being placed in a baby‐walker did not influence the time they spent interacting with their mother. Further, infants in the baby‐walker interacted significantly less than age‐matched walking infants. In line with Campos et al.'s 2000 proposal that onset of locomotion leads to changed exploration of the world, these findings support the idea that the shift to independent walking changes the child's interaction with those around them, leading, through development, to increased social behaviors.

### Early Gross Motor Abilities and Language in ASD

Evidence for a relationship between GM abilities and social‐communication in typical development has important implications for ASD, a neurodevelopmental disorder characterised by social‐communication impairments and restricted and repetitive behaviours (RRBs). While RRBs are part of the autistic triad, motor difficulties *per se* do not feature in the core diagnostic criteria. Although motor behaviors were once seen as being relatively intact in children with ASD [e.g. Gillberg et al., 1990] there is a growing consensus that motor development is atypical [e.g. see Bhat et al., 2011 for a review; Green et al., 2002; Green et al., 2009; Ozonoff et al., 2008]. Nevertheless, in comparison to those with developmental delay, Provost, Lopez, & Heimerl [2007] found no evidence for GM delays in toddlers with ASD. Early GM delays have also been documented in infants at risk for ASD [e.g. Landa & Garrett‐Mayer, 2006], although Leonard et al. [2014] found no autism specific impairments, only risk group differences.

Despite the fact that ASD is characterised by delayed language and nonverbal social communication difficulties [DSM‐5; American Psychiatric Association, 2013], only a few studies have investigated the relationship between motor ability and social communication in children with ASD. Hsu et al. [2004] found correlations of both GM and fine motor with concurrent expressive language as well as “social comprehension” and “personal social development” in 3‐year‐olds with ASD. Gernsbacher, Sauer, Geye, Schweigert, and Goldsmith [2008] used retrospective parental report of manual motor skill to predict current verbal fluency in children with ASD ranging from 2 to 18 years and found that manual skills, including clapping, pointing and turning a doorknob, significantly distinguished those classified with the low vs. high levels of speech fluency. However, it is important to note that these relationships could be driven in part by general intelligence or developmental level.

One recent study looking at motor abilities and rate of language development in infants at high familial risk for developing autism [Leonard et al., 2015] did control for general developmental level. Infants at risk, who have an older sibling with an autism diagnosis ‐ and thus have a 20% risk of developing autism themselves [Ozonoff et al., 2011] were prospectively followed throughout the first few years of life. Leonard et al. [2015] found that GM score in 7‐month‐olds predicted the subsequent rate of expressive, but not receptive, language in infants who went on to develop ASD. These results are consistent with Bhat et al. [2012], who found that early motor difficulties were related to poorer communication outcomes at 18 months in high‐risk infants.

### Walking and Language in ASD

In terms of walking, several studies have found evidence for atypical gait and postural stability in children with ASD compared with typically developing children [e.g. Damasio & Maurer, 1978; Jansiewicz et al., 2006; Minshew, 2004], although when compared with children with developmental delay, children with ASD show no significant difference in the onset of walking [Matson et al., 2010]. Kim [2008] examined whether onset of walking relates to later expressive and receptive language abilities in young children with ASD. In this study, no significant correlations were found between retrospective reports of motor milestones and current parent‐reported language (or indeed motor) functioning. It is unclear from Kim's [2008] method what exact question parents were asked with regard to onset of walking (e.g. whether it came from medical history or the autism diagnostic interview‐revised; ADI‐R).

The aim of this study was to investigate whether GM ability and age of walking onset predict subsequent receptive and expressive language trajectories between 2 and 9 years of age in children with ASD. The use of data from a large cohort of children followed longitudinally over multiple visits offers various advantages over previous studies, in particular the ability to model rate of language development. In addition, we controlled for general developmental level, taking account of floor effects in nonverbal IQ scores in this clinical population. We hypothesised that earlier walkers would show faster rates of language development, and that walking would remain a significant predictor even after controlling for general developmental level and overall GM ability.

## Method

### Participants

Participants come from the early diagnosis study, comprised of children seen in clinical research settings by Lord and coworkers over a number of years. Participants eligible for the study were consecutive referrals younger than 37 months from agencies across North Carolina and metropolitan Chicago serving young children with developmental delays. All 221 families agreed to participate in the study initially. One later withdrew and six other families became ineligible for inclusion when the children reached the age of 36 months before the first assessment could be scheduled. In addition, one child was excluded due to missing data on all measures and a further four were excluded owing to a subsequent cerebral palsy diagnosis. In this article, (see Table 1) data were analysed from 209 participants (170 male). There were 158 with an initial ASD or PDD‐NOS diagnosis (139 male; 94 from North Carolina state‐funded autism centers and 64 from a Chicago autism clinic) and 51 with general developmental delay (31 male). Children were assessed at 2, 3, 5, and 9 years of age.

**Table 1:** Descriptive Statistics: Age, VABS‐II expressive language (EL) and receptive language (RL) age equivalents and MSEL GM and VR subscale T‐scores

	2 years M (S.E.)	3 years M (S.E.)	5 years M (S.E.)	9 years M (S.E.)
Age in months	28.97 (0.36)	43.24 (0.44)	57.71 (0.80)	112.48 (1.17)
*N*	209	179	133	168
VABS RL AEs	12.14 (0.56)	20.94 (0.95)	31.63 (1.57)	54.75 (2.63)
*N*	208	178	129	159
VABS EL AEs	9.09 (0.47)	16.67 (0.72)	27.25 (1.64)	55.48 (3.37)
*N*	208	178	129	159
Regressed GM *T‐*score	37.09 (0.95)	–	–	–
*N*	194			
Regressed VR *T*‐score	31.46 (1.0)	–	–	–
*N*	191			

### Measures/Procedure

#### Vineland adaptive behavior scales (VABS‐II)

The VABS‐II [Sparrow, Cicchetti, & Balla, 2005] is a parent report measure of daily living skills. It was chosen because scores can be used across a range of ages and competency levels, and show high convergence with direct testing [Taylor, Pickering, Lord, & Pickles, 1998]. These properties are helpful when characterising language and communication trajectories in clinical populations [see Szatmari et al., 2009; Vos et al., 2014]. The current analyses use age equivalent (AE) scores from the receptive and expressive language subscales, which offer a more meaningful interpretation developmentally than raw scores. Caregivers report whether their child produces particular vocalisations or words (expressive language) or understands specific words or verbal information (receptive language) on a three‐point scale: “Never,” “Sometimes” or “Usually.”

#### Mullen scales of early learning (MSEL)

The Mullen scales of early learning (MSEL) is a standardised developmental assessment, which is used to assess early motor and cognitive development from 0 to 68 months. In this study, visual reception (VR) subscale scores are used as a proxy for nonverbal IQ. This subscale was chosen because, unlike the standard nonverbal IQ measure derived from both VR and fine motor scores, VR alone is not confounded with motor ability. The GM subscale score from the first (2‐year visit) was used as a measure of children's motor ability.

#### Autism diagnostic interview‐revised

The ADI‐R is a structured parent interview designed to distinguish children with ASD [Lord et al., 1994]. Algorithm scores comprise three subdomains: social behaviors, communication, and repetitive interests, as well as an overall total score. A toddler version of the ADI‐R, which includes additional items relating to the first 3 years of life, was given to 2‐ and 3‐year‐old children. In this analysis, the ADI‐R total score (total of all the algorithm items) was used as a covariate to account for level of autistic symptomatology. The onset of walking variable was taken from the ADI‐R item parent‐reported age of walking at the 2‐year visit. Parents were asked “At what age did [subject] walk without holding on?” and the age in months was recorded. For children who were not yet walking at this visit, the reported walking onset from the 3‐year visit was used. Data from children not walking by the 3‐year visit (*n* = 8) were coded as missing, in addition to missing data due to lack of ADI‐R completion (*n* = 7). Walking onset: mean = 14.79, S.E. = 0.42, *n* = 194.

#### Statistical analysis

In latent growth curve models (GCMs), both the intercept and slope of the regression equation are specified as latent variables, which can vary across individuals. In this article, we use GCMs to test whether parent‐reported “onset of walking” from the ADI‐R significantly predicted the rate of VABS‐II expressive and receptive language development from 2 to 9 years, while controlling for *MSEL* VR *T*‐score (a proxy for nonverbal IQ), severity of autism symptoms (ADI‐R total score) and *MSEL* GM *T*‐score. In the GCMs presented, we first ran a model with only walking onset as a predictor of language, second, we ran the analysis covarying for ADI‐R score and a VR T‐score with imputed values for children who had all been scored the basal score of 20 (71 children in this sample). To characterise the variability in scores at this low end of the scale we ran a regression in which we regressed the log of VR T‐scores on the log of VR AE scores and age at visit 2 to get the predicted values. The log scale was used to ensure predicted values were above zero. This gave regressed *T*‐scores for 191 children (see Table 1) because those with missing data for walking onset and language scores were excluded. These predicted values were then imputed in place of the baseline score of 20, giving *T*‐scores from 0 to 20 for these children. In model 3, GM *T*‐scores (with similarly imputed values for the 33 children with a baseline score of 20) were included as an additional predictor.

In the GCMs, the regression paths from the language scores to the slope factor were fixed to the chronological age of the child to account for the variability in the age around each visit (e.g. mean age at visit 1 was 29 months but children ranged from 13 to 38 months on different measures). This means that the intercept latent variable now represents expressive language score at age “0.” While the intercept is regressed on the predictor variables the results are not reported because language at “0” years is not interpretable. However, the intercept is allowed to correlate with the slope across all models, thus, implicitly controlling for baseline language level. All models were estimated using a robust maximum likelihood estimator (MLR) in Mplus [Muthén & Muthén, 2011] with the “*T‐*scores” option with analysis type = random. The MLR estimator accounts for missing data under the “missing at random” assumption, under which missingness is assumed to relate only to observed variables. It is more lenient than the standard “missing completely at random” assumption which can lead to biased estimates when listwise deletion is used. Correlations were estimated for the slope and intercept, as well as among the predictor variables (e.g. VR with onset of walking).

## Results

### Receptive Language (RL)

**Figure 1:**
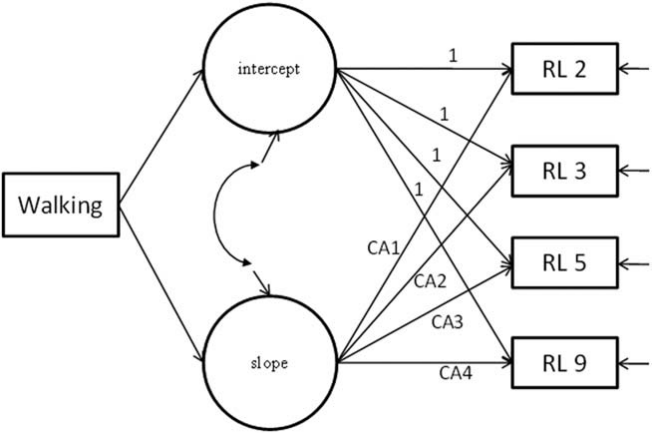
GCM for receptive language (RL) at four visits from 2 to 9 years, with the latent intercept and slope variables regressed on walking onset.

Results from a GCM (see Fig. 1) including only walking onset as a predictor (i.e. without inclusion of VR or ADI‐R total score) showed a significant relationship between walking and the slope of receptive language from 2 to 9 years (ß = −0.19, S.E. = 0.04, *P* < 0.001).

However, when we ran a GCM which included the ADI‐R score (ß = −0.08, S.E. = 0.05, *P* = 0.11) and the VR *T*‐score with imputed values to account for floor effects (ß = 0.15, S.E. = 0.02, *P* < 0.001), the relationship between walking onset and rate of receptive language development became nonsignificant (ß = −0.07, S.E. = 0.04, *P* = 0.11), although the coefficient was in the same direction.

**Figure 2:**
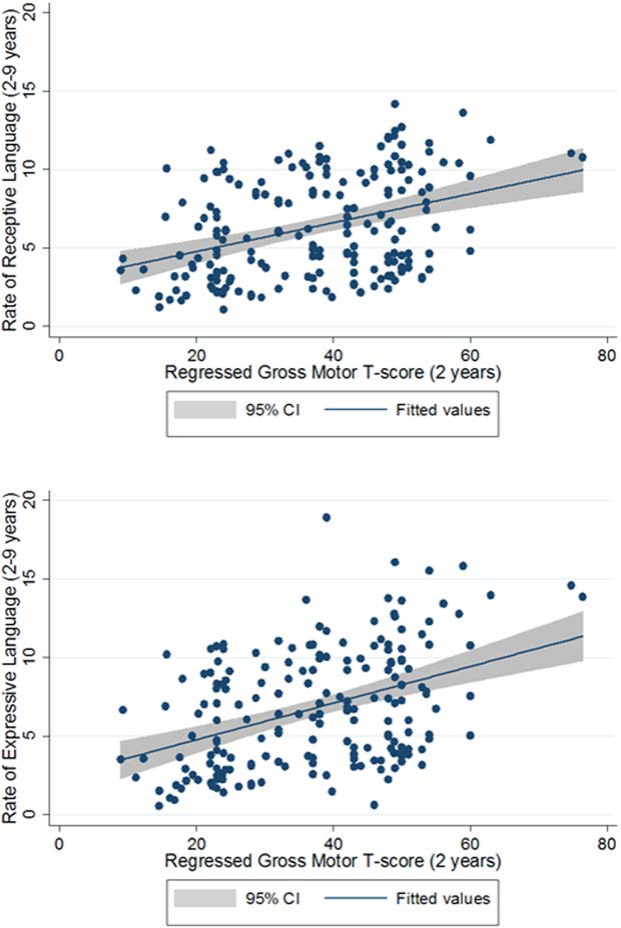
Gross motor ability predicts rate of receptive and expressive language growth between 2 and 9 years of age in children with ASD.

In Model 3, we also included GM *T*‐score with imputed values to account for floor effects. The relationship between walking and receptive language development dropped out of the model entirely (ß = −0.01, S.E. = 0.05, *P* = 0.79). Even after accounting for walking onset, VR score and ADI‐R score, GM skills still significantly predicted the slope of language development (ß = 0.06, S.E. = 0.03, *P* = 0.05; see Fig. 2).

### Expressive Language (EL)

For a GCM which included only walking as a predictor of expressive language rate, a significant relationship was found between walking onset and the slope of expressive language from 2 to 9 years (ß = −0.20, S.E. = 0.04, *P* < 0.001).

As with receptive language, when we ran a GCM for expressive language controlling for ADI‐R score (ß = −0.1, S.E. = 0.06, *P* = 0.06) and the imputed VR T‐score (ß = 0.17, S.E. = 0.02, *P* < 0.001), the relationship between walking and rate of language development became nonsignificant (ß = −0.07, S.E. = 0.04, *P* = 0.10).

When including GM (regressed *T‐*score) as a further predictor of expressive language rate, the relationship between walking and language rate was nonsignificant and essentially nil (ß = 0.00, S.E. = 0.05, *P* > 0.99). GM score did significantly predict language rate (ß = 0.09, S.E. = 0.04, *P* = 0.02) in this model even after controlling for walking onset, *MSEL* VR and autism symptoms.

### Outliers

Although children with known diagnosis of cerebral palsy were removed from the sample, there remained several late walkers. We reran the analysis removing participants reported to walk later than 36 months and results remained substantively similar. Model 1: walking remained a significant predictor of receptive (ß = −0.20, S.E. = 0.06, *P* = 0.001) and expressive language (ß = −0.22, S.E. = 0.06, *P* < 0.001). Model 2: The effect of walking on language became nonsignificant when accounting for nonverbal ability and autism symptoms (RL ß = −0.07, S.E. = 0.05, *P* = 0.17; EL ß = −0.08, S.E. = 0.06, *P* = 0.14). In Model 3, walking remained nonsignificant (RL ß = −0.009, S.E. = 0.06, *P* = 0.89; EL ß = 0.004, S.E. = 0.06, *P* = 0.95), while GM score remained a significant predictor of later language development (RL ß = 0.06, S.E. = 0.03, *P* = 0.05; EL ß = 0.09, S.E. = 0.04, *P* = 0.02).

## Discussion

This study aimed to determine whether GM abilities, specifically the age of walking onset, predicted subsequent rate of language development from 2 to 9 years in children referred for ASD. This article offers several key advantages over the previous literature by (1) using a large longitudinal sample of children with autism; (2) examining rate of change in language rather than language at a specific time point; (3) controlling for variability in nonverbal IQ and autistic symptoms; and (4) expanding the VR and GM *T*‐score measures to characterise the full range of variability at this lower end of the scale. Results showed that the age at which parents/caregivers report onset of walking in children with ASD predicts the subsequent rate of both receptive and expressive language development, but that this relationship does not hold after controlling for GM abilities, nonverbal IQ and severity of parent‐reported autistic symptomatology. However, GM abilities did remain a significant predictor for both receptive and expressive language development from 2 to 9 years.

The finding from Model 1—that later onset of walking is associated with slower language development in children with ASD—is consistent with findings from typical development, which suggest that walking onset is related to language development [Karasik et al., 2014; Oudgenoeg‐Paz, Volman, & Leseman, 2012; Walle & Campos, 2014]. The relationship between walking onset and language outcomes in children with ASD is also broadly consistent with the few studies looking at walking and language abilities in atypical development. For example, children with specific language impairment—a developmental language disorder—show a delay in the onset of walking [Haynes & Naidoo, 1991; Trauner, Wulfeck, Tallal, & Hesselink, 2000].

Various mechanisms have been proposed to underlie the relationship between walking and language, although at present much of our understanding, even in typical development, is somewhat speculative. One possibility is that having hands free to gesture and point allows the infant to engage in more frequent joint attention bids. Begus and Southgate [2012] showed that infants point interrogatively to request information, such as object names, which may increase the opportunity for word learning. While this would also apply to sitting, the change in posture from crawling to walking also enables infants to combine moving to an object of interest with orienting toward a person's face, potentially increasing joint attention. Joint attention bids predict subsequent linguistic ability in typically developing children [e.g. Carpenter, Nagell, & Tomasello, 1998; Morales et al., 2000] providing a “scaffold” for emerging communication [Baker & Nelson, 1984].

Joint attention is also one of the earliest discriminators of autism [Charman, 2003], and predicts both concurrent and longitudinal language abilities in autistic children [e.g. Charman et al., 2003; Sigman & Ruskin, 1999; Siller & Sigman, 2008]. The effect of delayed walking on language in autism may be compounded across development, as joint attention abilities remain atypical, rather than simply being delayed. However, it is important to note that we do not see joint attention deficits in developmentally delayed children, despite GM delays [Shumway & Wetherby, 2009; Watson, Crais, Baranek, Dykstra, & Watson, 2013]. While it may be that, in autism, very early atypicalities in gaze following behavior interact with GM development, future research will be required to establish the precise nature of any association: whether joint attention delays in autism could mediate the observed relationship between walking and language development, and whether effects are additive or multiplicative [Bedford et al., 2014].

In Model 2, a proxy for nonverbal IQ which taps memory and attention, the *MSEL* regressed VR *T*‐score, was controlled for in the analysis. ADI‐R symptoms of autism were also covaried, because the participant group was particularly heterogeneous, containing children initially classified as having developmental delay, pervasive developmental disorder and autism. ADI‐R symptoms also relate to general development, and in this model ADI‐R was negatively correlated with VR scores (*P*‐values < 0.009). Thus, while the relationship between walking and language was reduced, with the significance level becoming marginal (*P*‐values = 0.1), this model is quite strict in its control of general developmental level.

The results illustrate the importance of controlling for general ability when looking at the relationship between motor and language skills. The floor effects in the *MSEL* VR measure also emphasise the need for measures which better capture variability at this low end of the distribution [see Farmer, Golden, & Thurm, 2015]. The latter point is of particular relevance to clinical populations. While the method we used to regress the VR score against AE score may be overly attributing variability between children at the end of the scale, clearly giving everyone a baseline score of 20 loses interesting variability within these children.

In the final and most stringent model, when we included GM and VR regressed *T*‐scores together with a measure of autism symptom severity, the relationship between walking and language became nonsignificant. However, consistent with previous studies in autism [Gernsbacher et al., 2008; Hsu et al., 2004] GM skills did significantly predict later language development. GM abilities are amongst the earliest skills infants develop. Characterising such predictors of reduced language growth that are measureable early in development has important clinical implications in terms of earlier identification and potential intervention. While motor delays are not specific to autism, infants and toddlers who are already at risk for communication difficulties may be more greatly affected by early motor delays. This is consistent with work by Viholainen, Ahonen, Cantell, Lyytinen and Lyytinen [2002], which found that children at familial high‐risk for dyslexia who also had delayed GM development subsequently showed a reduced vocabulary size and slower reading speed.

While understanding the precise mechanisms underlying the relationship between GM and language is beyond the scope of this study, it is possible that neurological development of the cerebellum may play a role in explaining the relationship [Diamond, 2000; Walle & Campos, 2014], as it shows activation both during motor learning tasks and cognitive tasks [Diamond, 2000]. Another possible mediating variable is multisensory integration, which is likely to increase at the onset of motor milestones due to a tighter coupling of proprioceptive and visual information. Multisensory integration is also thought to play an important role in language development, from the canonical babbling stage [Iverson, 2010] through to adult audio‐visual integration for speech perception [e.g. McGurk & McDonald, 1976]. Iarocci and McDonald [2006] have argued that difficulties in multisensory speech perception offer a useful way to conceptualise sensory processing in autism, although evidence for a deficit is mixed [DeGelder, Vroomen, & Van der Heide, 1991; Foss‐Feig et al., 2010; Taylor, Issac, & Milne, 2010; Williams, Massaro, Peel, Bosseler, & Suddendorf, 2004].

It is also important to note that the *MSEL* GM scale includes many items relating to walking ability, and may offer a better characterisation of walking than parental report of age of onset. Within the current study it is, thus, not possible to tease apart the relative importance of different aspects of GM skill, such as sitting and crawling, which also alter the way a child interacts with the environment. Future studies are needed to test the specificity of different GM milestones in contributing to language development.

A particular strength of this study is the use of different parent report and observational measures (i.e. ADI‐R, VABS‐II, and *MSEL*) to assess abilities in a large cohort of children with ASD. However, an important limitation of these measures is the level of detail they provide. While the VABS‐II is a well validated measure of receptive and expressive language, a questionnaire such as the MacArthur‐Bates communicative development inventory [Fenson et al., 1993] would enable more detailed measurement of children's vocabulary. Perhaps more importantly, using retrospective parent report to assess walking onset does not capture the variability in learning to walk assessed in longitudinal, prospective studies of typical development [e.g. Clearfield et al., 2008; Karasik et al., 2011]. Again, by following infants at high risk for autism, more fine‐grained assessment of motor milestones will be possible. The notion of “milestones” implies discontinuity (e.g. from not walking to taking a first step). However, in reality the process of learning to walk is likely to develop over a period of days or even weeks from first steps through to confident walking. Studying this *as it emerges* will give us a better understanding of the underlying mechanisms and likely bidirectional links between motor and language abilities.

In conclusion, this study found evidence for a relationship between early GM abilities and the subsequent rate of receptive and expressive language development in children with autism. As well as looking at overall GM abilities, we also aimed to test the specific hypothesis that walking onset may be the primary motor milestone of importance. However, although walking onset was a significant predictor in a simple model, the effect did not remain significant after accounting for nonverbal IQ and overall GM abilities. To elucidate the underlying mechanisms, future prospective studies will be required to establish the relative importance of specific motor milestones such as crawling and sitting to language development.
